# Diagnostic Accuracy of Metagenomic Next-Generation Sequencing in Sputum-Scarce or Smear-Negative Cases with Suspected Pulmonary Tuberculosis

**DOI:** 10.1155/2021/9970817

**Published:** 2021-09-03

**Authors:** Ning Zhu, Daibing Zhou, Shengqing Li

**Affiliations:** Department of Pulmonary and Critical Care Medicine, Huashan Hospital, Fudan University, Shanghai, China 200040

## Abstract

**Objective:**

To investigate the diagnostic accuracy of metagenomic next-generation sequencing (mNGS) in bronchoalveolar lavage fluid (BALF) samples or lung biopsy specimens from which suspected pulmonary tuberculosis (PTB) patients have no sputum or negative smear.

**Materials and Methods:**

Sputum-scarce or smear-negative cases with suspected PTB (*n* = 107) were analyzed from January 2018 to June 2020. We collected BALF or lung tissue biopsy samples with these cases of suspected TB during hospitalization. The diagnostic accuracy of mNGS for these samples was compared with those of conventional tests or the T-SPOT.TB assay.

**Results:**

46 cases of PTB patients and 61 cases of non-PTB patients were finally enrolled and analyzed. mNGS exhibited a sensitivity of 89.13%, which was higher than conventional tests (67.39%) but equivalent to those of the T-SPOT.TB assay alone (76.09%) or T-SPOT.TB assay in combination with conventional tests (91.30%). The specificity of mNGS was 98.36%, similar to conventional tests (95.08%) but significantly higher than those of the T-SPOT.TB assay alone (65.57%) or the T-SPOT.TB assay in combination with conventional tests (63.93%). There was no significant difference in the diagnostic accuracy of mNGS in BALF samples and lung biopsy tissue specimens.

**Conclusion:**

Our findings demonstrate that mNGS could offer improved detection of Mycobacterium tuberculosis in BALF or lung tissue biopsy samples in sputum-scarce or smear-negative cases with suspected PTB.

## 1. Introduction

Tuberculosis remains a major global health problem that causes ill health to millions of people worldwide every year. *M. tuberculosis* (*M*. *tb*) is the aetiology. Pulmonary tuberculosis (PTB) is the most common form of tuberculosis, accounting for 85% and 70% of HIV-negative and HIV-positive tuberculosis cases, respectively [1]. Left untreated, disseminated tuberculosis, including subclinical PTB [2], is potentially fatal. Therefore, early and timely diagnosis of tuberculosis is critical to preventing disease progression and improving prognosis [3].

Recently, standard diagnostic methods for tuberculosis include acid-fast staining, tuberculosis culture, GeneXpert MTB/RIF assay, and T-SPOT.TB assay. Although the sensitivity of microscopic examination of acid-fast bacilli (AFB) in sputum specimens is 50% or less, AFB staining is still the most widely used rapid diagnostic method for tuberculosis. However, its value for patients who cannot produce sputum spontaneously is very little [4]. Accordingly, the World Health Organization (WHO) recommends mycobacterial culture, which exhibits a high sensitivity for detecting *M*. *tb*, as the diagnostic gold standard. Unfortunately, due to the slow growth of *M*. *tb*, mycobacterial culture cannot meet the clinical needs of the diagnosis and treatment of TB. Moreover, mycobacterial culture requires extensive laboratory infrastructure, limiting its application in resource-limited settings. The T-SPOT.TB assay is an auxiliary assay commonly used in the diagnosis of PTB. The sensitivity (78.4%) and specificity (59.0-93.0%) of this method are reportedly superior to those of conventional methods [5–7]. However, the results of the T-SPOT.TB assay can be affected by age, BMI, immune status, and other factors [8, 9]. New diagnostic tools-urine lipoarabinomannan assay has been excavated appropriately, but more evidence needs to be demonstrated [10]. Since sputum-scarce or smear-negative PTB patients account for a large proportion of cases, a quick and accurate diagnosis of PTB is a challenge to clinicians.

Metagenomic next-generation sequencing (mNGS) is a new technology for detecting microorganisms and allows thousands to billions of DNA fragments to be independently sequenced simultaneously. Compared with conventional tests, mNGS is fast and accurate and has high throughput. mNGS can also detect unculturable pathogens and those that are difficult to cultivate without culture and retrieve all DNA without bias to identify and type pathogenic microorganisms. mNGS can detect pathological organisms from specimens such as bronchoalveolar lavage fluid (BALF) [11], cavity effusion, cerebrospinal fluid, sputum, urine, and blood [12]. In addition, mNGS could be used to rapidly identify pathogens during severe [11], mixed [13], and rare pathogen infections [14]. It was reported by Miao et al. that the sensitivity and specificity of mNGS in the diagnosis of infectious diseases are 50.7% and 85.7%, respectively [15]. Li et al. found that, compared with culture methods, mNGS had a diagnostic sensitivity of 88.89% and a specificity of 74.07% in diagnosing severe respiratory diseases [11]. To date, few studies have evaluated the diagnostic accuracy of mNGS for PTB with negative sputum smears, highlighting the need for further research. In this study, we analyzed the diagnostic accuracy of mNGS in sputum smear-negative patients with PTB.

## 2. Materials and Methods

### 2.1. Study Population and Design

A retrospective study of adult patients with suspected PTB was enrolled from Huashan Hospital, Fudan University, between January 1, 2018, and June 30, 2020. All the enrolled patients were required to meet one of the following criteria: (1) no sputum or sputum scarcely and (2) negative sputum/acid-fast bacilli smear and *M. tb* culture at least twice. Suspected PTB referred to those patients meeting the following criteria (at least one item): (1) defined TB exposure; (2) performing symptoms of tuberculosis poisoning, such as subacute cough, fever, night sweat, and loss of weight; and (3) chest X-ray indicates miliary pulmonary nodules or patchy shadows.

Clinicians ultimately diagnosed PTB cases according to the clinical guidelines of the Ministry of Health of the People's Republic of China. Defined PTB cases should comply with any of the following criteria: (1) The microbiological results (including acid-fast staining smear or culture of tuberculosis in BALF specimen) were positive. (2) The pathological result of lung biopsy tissue is consistent with the pathological features of tuberculosis. (3) The lung lesions reduced or disappeared after three months of antituberculosis treatment. Otherwise, they are classified as non-PTB cases. The non-PTB cases were treated with antibiotics such as penicillin and cephalosporins and were followed up for at least three months.

### 2.2. BALF and Lung Biopsy Specimens

Bronchoscopic alveolar lavage or computerized tomography-guided percutaneous lung biopsy was performed in the lung lesions. Each specimen was divided into two parts for mNGS analysis or conventional tests. All enrolled patients signed informed consent before undergoing bronchoscopy tests or lung puncture.

BALF was obtained through bronchoscopy from the lung lesions according to the standard procedure. Briefly, 60 mL-100 mL saline was injected into the segmental bronchus and withdrawn after a brief wash. The qualified BALF (at least 20 mL) was divided in two and tested by conventional methods, including the acid-fast staining smear and culture for *M. tb*, or placed in a sterile sputum container, stored at -20°C, and sent to BGI Genomics (Shenzhen, China) for testing. Similarly, lung tissue specimens were obtained by computerized tomography-guided percutaneous lung biopsy. Each lung tissue specimen was divided into two parts for mNGS analysis and pathological tests. Acid-fast staining smear or mycobacterial culture of BALF and pathological detection of lung biopsy tissue were classified as conventional tests. If any one of these tests was positive, the conventional tests were considered to be positive. Meanwhile, if all of them were negative, the conventional tests were considered to be negative.

### 2.3. T-SPOT.TB Assay

If these patients with no sputum or negative smear have been characterized of plausible TB-related symptoms or radiological features, we collected peripheral venous blood for T-SPOT.TB assay (Oxford Immunotec, Medical Device Co., Ltd., Shanghai, China). The T-SPOT.TB assay belongs to interferon-gamma release assays (IGRAs) and is based on the release of interferon-gamma secreted by activated effector T cells, both CD4^+^ and CD8^+^, when stimulated by TB-specific antigens. According to the protocol, briefly, peripheral blood mononuclear cells (PBMCs) were isolated from blood specimens using heparin lithium-anticoagulant tubes. The cells were washed, counted, and normalized to prepare a recommended cell suspension. A recommended number of PBMCs were added into four designed microwells (positive control wells, A wells: ESAT-6, B wells: CFP-10 and negative control wells) and stimulated with TB-specific antigen (ESAT-6 and CFP-10) and incubated at 37°C for 24 hours. A detection reagent was added and reacted with the second antibody. The reaction produced spots. Spots were then counted. The criteria of positive results interpretation were below: (1) if the number of spots in the negative control well was no more than 5, and the positive spots in A or B wells were more than 6 spots compared than that of negative control wells and (2) if the spots in the negative control wells were 6 to 10, and the positive spots in A or B wells were more than two times that of the negative control. Results were considered negative if the spots did not conform to the criteria above and the positive control wells were normal. In addition, it was supposed to be inconclusive if the number in negative control wells was more than 10, then the test of the second sample is necessary.

### 2.4. mNGS and Data Analyses

DNA extraction, library construction, and sequencing were performed as previously described [15, 16]. All specimens were promptly stored in sterile pipes for following examinations. Briefly, 1 mL BALF samples or 0.5 g tissue samples from each patient were collected and subjected to DNA extraction using TIANamp Micro DNA Kit (DP316, TIANGEN Biotech). DNA libraries were constructed by DNA fragmentation, end-repair, adapter ligation, and PCR amplification and subjected to quality control analysis using Agilent 2100 bioanalyzer. Quality verified libraries were sequenced by the BGISEQ-50 platform (BGI Genomics, Shenzhen) [17]. After filtering low-quality reads, human host sequences, and low-complexity reads, taxonomy assignment was conducted by sequence alignment using Burrows-Wheeler Alignment [18] to reference databases. They were strictly compared with the homologous sequences of H37Rv strain (NCBI Reference Sequence: NC_000962.3) [19], consisting of 4,945 whole-genome sequences of viral taxa, 6350 bacterial genomes or scaffolds, 1064 fungi related to human infection, and 234 parasites associated with human diseases. *M*. *tb* is an intracellular pathogen with low abundance and low possibility of pollution, the detection threshold of *M. tb* should be set low. So, mNGS is considered positive when at least 1 read is mapped to the species or genus level. Otherwise, mNGS was considered negative [20].

### 2.5. Statistical Analysis

Continuous variables are expressed as mean ± S.D.2 × 2 contingency tables were derived to determine true positives, true negatives, false negatives, false positives, sensitivity, specificity, positive predictive value (PPV), and negative predictive value (NPV). A detection test was positive in the case of defined PTB, which was considered to be true positive. In the case of defined non-PTB, it was considered to be false positive. On the contrary, a detection test is negative in determining defined non-PTB, which is considered to be true negative; Negative cases in defined PTB were considered false negative. All statistics are reported as absolute values with their 95% confidence interval (95% CI). Paired McNemar chi-square test was used to compare the diagnostic accuracy of mNGS and conventional tests or T-SPOT.TB assay. A 2-sided *p* value < 0.05 was considered statistically significant. All statistical analyses were performed using SPSS 22.0 (IBM, Armonk, NY, USA). We used MedCalc software (MedCalc Software Ltd.) for ROC curve analysis among different groups.

## 3. Results

### 3.1. Clinical Characteristics of the Participants

**Figure 1:**
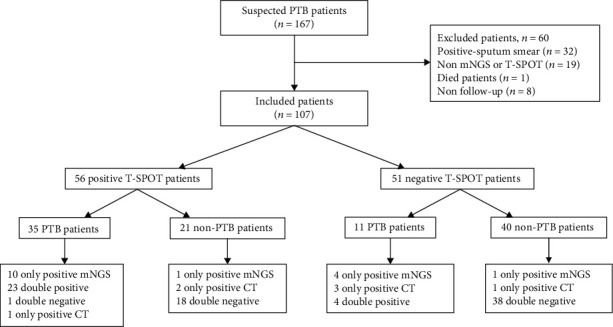
Classification of the inclusion criteria for patients based on the T-SPOT.TB assay.

A total of 167 suspected PTB patients between January 1, 2018, and June 30, 2020, were initially included in our study. Sixty patients were subsequently excluded, 32 of whom had positive sputum smears, 19 had no mNGS or T-SPOT.TB assay data, 8 had no follow-up, and one died without a definite diagnosis. Consequently, 107 sputum-scarce or smear-negative patients with suspected PTB were finally enrolled in the study (Figure 1). Since the T-SPOT.TB assay is a commonly used auxiliary assay in the diagnosis of PTB, and each enrolled patient was subjected to the T-SPOT.TB assay. All enrolled patients underwent bronchoscopy or percutaneous lung biopsy during hospitalization. BALF or lung tissue specimens were obtained for testing by either conventional tests or mNGS.

Among the enrolled patients, 46 cases were diagnosed with PTB, and 61 cases were diagnosed with non-PTB. There were significant differences in age, body mass index (BMI), and the T-SPOT.TB assay positivity rates between the PTB and non-PTB groups. The average age in the PTB group was significantly lower than in the non-PTB group (52.09 ± 17.19 vs. 58.66 ± 14.76; *p* < 0.001). Similarly, the BMI in the PTB group was markedly lower than in the non-PTB group (21.09 ± 2.97 vs. 23.56 ± 4.70; *p* < 0.001). It indicated that age and BMI were the risk factors of PTB. The positivity rate of the T-SPOT.TB assay in the PTB group was significantly higher than in the non-PTB group (*p* < 0.001). It suggested that routine PTB screening in patients with positive T-SPOT.TB assay is necessary. No significant difference was observed in other clinical factors, such as gender, smoking history, glucocorticoid usage, and lung abnormalities on chest radiograph. (Table 1).

### 3.2. Diagnostic Performance of mNGS, Conventional Tests, and the T-SPOT.TB Assay in PTB

The performance of mNGS was compared with that of conventional tests and the T-SPOT.TB assay in the diagnosis of PTB. These results showed that mNGS was more sensitive than conventional tests (89.13%, 95% CI: 75.64, 95.93 vs. 67.39%, 95% CI: 51.86, 80.03; *p* < 0.05). Although the sensitivity of mNGS was higher than that of the T-SPOT.TB assay, there was no statistically significant difference between them (89.13%, 95% CI: 75.64, 95.93 vs. 76.09%, 95% CI: 60.90, 86.92; *p* = 0.099). In addition, the sensitivity of mNGS was equivalent to that of conventional pathogen detection methods in combination with the T-SPOT.TB assay (89.13%, 95% CI: 75.64, 95.93 vs. 91.03%, 95% CI: 78.31, 97.18; *p* = 0.726).

The specificity of mNGS was similar with that of conventional tests (98.36%, 95% CI: 90.02, 99.91 vs. 95.08%, 95% CI: 85.40, 98.72; *p* = 0.611). The specificity of the T-SPOT.TB assays was significantly lower than that of mNGS (65.57%, 95% CI: 52.22, 76.96 vs. 98.36%, 95% CI: 90.02, 99.91; *p* < 0.001). Meanwhile, compared with conventional tests in combination with the T-SPOT.TB assay, mNGS exhibited significantly higher specificity (98.36%, 95% CI: 90.02, 99.91 vs. 63.93%, 50.57, 75.54; *p* < 0.001).

The PPV and NPV of mNGS were 97.62% and 92.31%, respectively, whereas the PPV and NPV of conventional tests were 91.18% and 79.45%, respectively. By comparison, the PPV and NPV of the T-SPOT.TB assay were 62.50% and 78.43%, respectively. However, the PPV and NPV of conventional tests in combination with the T-SPOT.TB assay were 65.63% and 90.70%, respectively (Table 2).

**Figure 2:**
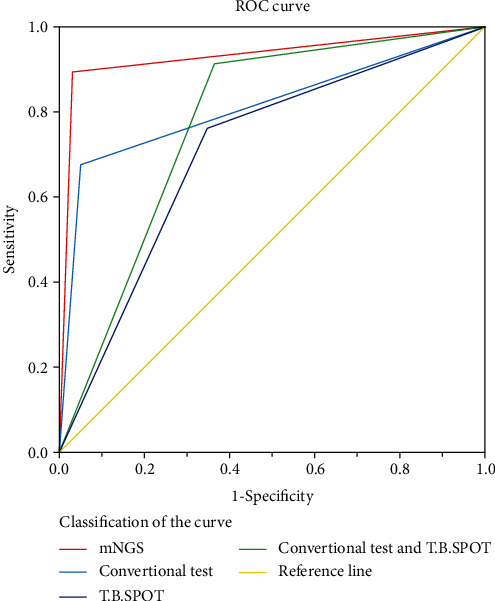
The diagnostic accuracy of different detection techniques.

The area under the curve (AUC) for mNGS (0.929, 95% CI:0.870, 0.988) was larger than that of the other detection tests, which were 0.812, 95% CI: 0.722, 0.902 for conventional tests, 0.708, 95% CI: 0.608, 0.808, for the T-SPOT.TB assays, and 0.776, 95% CI: 0.687, 0.866 for conventional tests in combination with the T-SPOT.TB assays (Figure 2). So, compared with conventional tests or the T-SPOT.TB assays, mNGS has an improved diagnostic accuracy in suspected PTB.

### 3.3. Diagnostic Performance of mNGS in BALF and Lung Tissue Biopsy Specimens

Among the enrolled patients, 78 underwent BALF collection, including 32 patients with PTB and 46 patients without PTB. Twenty-nine patients underwent lung lavage biopsy, including 14 PTB patients and 15 non-PTB patients.

The sensitivity of mNGS in BALF samples was similar with that of lung tissue biopsy specimens (90.63%, 95% CI: 73.83, 97.55 vs. 85.71%, 95% CI: 56.15, 97.47; *p* = 0.633). Meanwhile, there was no significant difference between the specificity of BALF or lung biopsy tissue specimens (97.83%, 95% CI: 87.03, 99.89 vs. 93.33%, 95% CI: 66.03, 99.65; *p* = 0.989). The PPV and NPV of mNGS in BALF samples were 96.67% and 93.75%, respectively, whereas the PPV and NPV of mNGS in lung tissue biopsy specimens were 92.31% and 87.5%, respectively (Table 3). These results indicated that the diagnostic accuracy of mNGS was independent of the sample type. Therefore, BALF and lung tissue biopsy specimens can be replaced with each other during the diagnosis of tuberculosis, especially where there are contraindications.

## 4. Discussion

Mycobacterium tuberculosis is an ancient bacterium and could cause pulmonary infection. Despite the typical vaccine application and a recommended four-drug treatment, M. tuberculosis infection also occurs worldwide, especially in the poor and developing countries. Significantly, HIV infection and insufficient knowledge of incipient, and subclinical TB infection may accelerate TB-related disease spread [2, 21]. Subclinical TB infections, known as the early staging of active TB disease, are easily ignored because of no symptoms. Although existing radiologic or microbiologic assays could help determine the patients [21, 22], diagnosing PTB in sputum-scarce or smear-negative patients is especially difficult in limited-resource environments. Consequently, it can often be impossible for some clinicians to prescribe early empirical treatment for suspected PTB based on the appearance of miliary patterns on chest X-rays, which cannot replace etiological confirmation. Therefore, clinicians have to resort to other diagnostic techniques for sputum-scarce or smear-negative patients with suspected PTB.

For infectious pulmonary lesions, the most common specimens include sputum, BALF, and lung tissue biopsy [23–25], and to obtain high-quality respiratory samples from suspected sputum-scarce or smear-negative tuberculosis patients, BALF and lung biopsy are mainly used [26, 27]. In our study, BALF or lung tissue biopsy samples were obtained from these suspected TB patients during hospitalization, who had no sputum or negative sputum smear.

The rapid development of mNGS has stimulated detection of bacteria, fungi, viruses, and parasite sequences by nontargeted DNA/RNA sequencing, facilitating the quick identification of pathogens. Thus, mNGS has been widely applied to improve the diagnosis of various infectious diseases. For example, Huang et al. used mNGS to detect microbes related to human diseases in 94.49% of samples from patients with pulmonary infections who had received negative results from traditional pathogen detection methods, indicating that the accuracy and sensitivity of mNGS are higher than those of standard pathogen detection techniques [28]. Similarly, Wang et al. found that mNGS was more sensitive than traditional methods in mixed pulmonary infections [13] whereas Chen et al. found that the results obtained using mNGS in BALF of patients with severe disease were highly consistent with those of culture [29].

The diagnostic value of mNGS for sputum-scarce or smear-negative PTB was evaluated in our study. Compared to conventional tests, mNGS was more sensitive, but its specificity was similar to conventional tests. Compared to conventional tests in combination with the T-SPOT.TB assay, the specificity of mNGS was much higher, but the sensitivity of mNGS was equivalent to that of conventional tests in combination with the T-SPOT.TB assay. The area under the curve (AUC) for mNGS was larger than that of conventional tests or the T-SPOT.TB assays. It indicated that mNGS had an improved diagnostic performance in suspected PTB. To determine the utility of mNGS for tuberculosis diagnosis in different sample types, we further evaluated the performance of mNGS in BALF and lung tissue biopsy samples. The results demonstrated that the diagnostic efficiency of mNGS in BALF and lung biopsy was highly consistent. Thus, if one sampling technique is contraindicated, clinicians can choose the alternative technique without hesitation. Therefore, these results suggested that mNGS could offer improve diagnostic detection in sputum-scarce or smear-negative PTB, and BALF and lung tissue biopsy tissue specimens can be used interchangeably.

However, our study had some limitations. Firstly, our study was limited by a relatively small sample size, which might have introduced bias in our interpretation of the data, and further studies to evaluate the diagnostic efficiency of mNGS are required. Secondly, since our research used BALF or lung tissue puncture specimens, the location of the lavage or puncture might not have contained pathogenic bacteria, resulting in false negative results. Meanwhile, some colonization bacteria could also be detected in specimens. so, mNGS is still an auxiliary tool that needs to be integrated with clinical feature, radiological imaging, and laboratory tests. Thirdly, we could not rule out false negative and false positive effects in mNGS data, which can occur if (1) the sequence depth is not enough; (2) the host genome background is high, and the microbial pathogen biomass is low; (3) antibiotics are used before detection; and (4) microbial genome pollution in environment or human flora [28, 30].

In summary, our findings demonstrate that mNGS could offer improved detection of Mycobacterium tuberculosis in BALF or lung tissue biopsy samples in sputum-scarce or smear-negative cases with suspected PTB.

## Figures and Tables

**Table 1:** Clinical characteristics of enrolled patients.

	Number	PTB	Non-PTB	*p* value
Number of patients	107	46	61	
Age (years) (mean ± SD)		52.09 ± 17.19	58.66 ± 14.76	<0.001
Gender				0.229
Male, *n* (%)	56	21 (45.65)	35 (57.38)	
Female, *n* (%)	51	25 (54.35)	26 (42.62)	
BMI (mean ± SD)		21.09 ± 2.97	23.56 ± 4.70	<0.001
Male	56	21.39 ± 3.38	24.72 ± 4.92	<0.001
Female	51	20.84 ± 2.54	21.79 ± 3.71	<0.001
Smoking history				0.205
Smoker	25	8	17	
Nonsmoker	82	38	44	
T-SPOT.TB assay				<0.001
Positive	56	35	21	
Negative	51	11	40	
Comorbidities				0.365
Yes	28	10	18	
No	79	36	43	
Glucocorticoid usage				0.383
Yes	15	8	7	
No	92	38	54	
Abnormality on chest radiograph				0.801^∗^
Upper lobe of left lung	23	12	11	
Lower lobe of left lung	18	7	11	
Upper lobe of right lung	29	13	16	
Middle lobe of right lung	14	5	9	
Lower lobe of right lung	22	8	14	
Hilum pulmonale	1	1	0	

^∗^Comparison between the left and upper and lower right and middle right lobe.

**Table 2:** Diagnostic performance of mNGS, conventional tests, and the T-SPOT.TB assay in PTB.

	PTB	Non-PTB	Sensitivity (95% CI)	Specificity (95% CI)	PPV (95% CI)	NPV (95% CI)
mNGS			89.13 (75.64, 95.93)	98.36 (90.02, 99.91)	97.62 (85.91, 99.88)	92.31 (82.25, 97.13)
Positive	41	1				
Negative	5	60				
Conventional tests			67.39 (51.86, 80.03)	95.08 (85.40, 98.72)	91.18 (75.19, 97.69)	79.45 (68.07, 87.68)
Positive	31	3				
Negative	15	58				
T-SPOT.TB assay			76.09 (60.90, 86.92)	65.57 (52.22, 76.96)	62.50 (48.52, 74.77)	78.43 (64.30, 88.25)
Positive	35	21				
Negative	11	40				
Conventional tests combined with T-SPOT.TB assay			91.30 (78.31, 97.18)	63.93 (50.57, 75.54)	65.63 (52.61, 76.75)	90.70 (76.95, 96.98)
Positive	42	22				
Negative	4	39				

Abbreviations: mNGS: metagenomic next-generation sequencing; PTB: pulmonary tuberculosis; Non-PTB: non-pulmonary tuberculosis; PPV: positive predictive value; NPV: negative predictive value.

**Table 3:** Diagnostic performance of mNGS in BALF and lung tissue biopsy specimens.

mNGS	PTB	Non-PTB	Sensitivity (95% CI)	Specificity (95% CI)	PPV (95% CI)	NPV (95% CI)
BALF			90.63 (73.83, 97.55)	97.83 (87.03, 99.89)	96.67 (80.95, 99.83)	93.75 (81.80, 98.37)
Positive	29	1				
Negative	3	45				
Lung biopsy specimens			85.71 (56.15, 97.49)	93.33 (66.03,99.65)	92.31 (62.09, 99.60)	87.5 (60.41, 97.80)
Positive	12	1				
Negative	2	14				

Abbreviations: mNGS: metagenomic next-generation sequencing; BALF: bronchoalveolar lavage fluid; PTB: pulmonary tuberculosis; Non-PTB: non-pulmonary tuberculosis; PPV: positive predictive value; NPV: negative predictive value.

## Data Availability

The data used to support the findings of this study are included within the supplementary information file(s).

## Supplementary Materials

Supplementary Materials Supplementary Table: clinical characterize of 51 negative T-SPOT patients included. Clinical characterize of 56 positive T-SPOT patients included.
