# Supplementary material for: Diagnostic Accuracy of Metagenomic Next-Generation Sequencing in Sputum-Scarce or Smear-Negative Cases with Suspected Pulmonary Tuberculosis
Source: Biomed Res Int. 2021 Sep 3;2021:9970817. doi: 10.1155/2021/9970817 (PMC8437628; doi:10.1155/2021/9970817)
Supplement: Supplementary Materials — Supplementary Table: clinical characterize of 51 negative T-SPOT patients included. Clinical characterize of 56 positive T-SPOT patients included. [file 9970817.f1.docx]

| **Supplementary Table.**  **Clinical characterize of 51 negative T-SPOT patients included.** | | | | | | | | | | | | | |
| --- | --- | --- | --- | --- | --- | --- | --- | --- | --- | --- | --- | --- | --- |
|  | **Gender** | **Age** | **Height** | **Weight** | **BMI** | **Smoking history** | **lesion** | **Pathological diagnose** | **Metagenomic next generation sequencing** | **BALF smear** | **Mycobacterium tuberculosis (M. tb)** | **Metagenomic next generation sequencing** | **conventional methods** |
| 1 | Female | 29 | 160 | 45 | 17.6 | Non smoking | Upper lobe of right lung | No | BALF+ | - | Yes | Positive | Negative |
| 2 | Female | 39 | 155 | 60 | 25.0 | Non smoking | Upper lobe of right lung | TB | BALF+ | + | Yes | Positive | Positive |
| 3 | Female | 30 | 158 | 49 | 19.6 | Non smoking | Upper lobe of right lung | TB | lung biopsy- | - | Yes | Negative | Positive |
| 4 | Female | 67 | 165 | 58 | 21.3 | Non smoking | Upper lobe of right lung | TB | BALF+ | 1+ | Yes | Positive | Positive |
| 5 | Female | 65 | 155 | 64 | 26.6 | Non smoking | Upper lobe of right lung | TB | BALF+ | + | Yes | Positive | Positive |
| 6 | Female | 73 | 165 | 54 | 19.8 | Non smoking | Upper lobe of right lung | No | BALF+ | - | Yes | Positive | Negative |
| 7 | Female | 62 | 165 | 56 | 20.6 | Non smoking | Upper lobe of right lung | TB | lung biopsy+ | - | Yes | Positive | Positive |
| 8 | Female | 71 | 150 | 52.4 | 23.3 | Non smoking | Lower lobe of right lung | TB | lung biopsy+ | 2+ | Yes | Positive | Positive |
| 9 | Female | 39 | 163 | 60 | 22.6 | Non smoking | Lower lobe of right lung | No | BALF+ | - | Yes | Positive | Negative |
| 10 | Female | 26 | 168 | 54 | 19.1 | Non smoking | Lower lobe of right lung | No | BALF+ | - | Yes | Positive | Positive |
| 11 | Female | 42 | 168 | 62 | 22.0 | Non smoking | Lower lobe of right lung | TB | BALF+ | 2+ | Yes | Positive | Positive |
| 12 | Female | 73 | 160 | 45 | 17.6 | Non smoking | Middle lobe of right lung | No | BALF+ | 2+ | Yes | Positive | Positive |
| 13 | Female | 58 | 155 | 49 | 20.4 | Non smoking | Middle lobe of right lung | TB | lung biopsy+ | - | Yes | Positive | Negative |
| 14 | Female | 32 | 157 | 55.5 | 22.5 | Non smoking | Upper lobe of left lung | No | BALF+ | - | Yes | Positive | Positive |
| 15 | Female | 29 | 169 | 56 | 19.6 | Non smoking | Upper lobe of left lung | TB | lung biopsy+ | - | Yes | Positive | Negative |
| 16 | Female | 31 | 159 | 41 | 16.2 | Non smoking | Upper lobe of left lung | TB | lung biopsy+ | - | Yes | Positive | Positive |
| 17 | Female | 53 | 162 | 59 | 22.5 | Non smoking | Upper lobe of left lung | No | BALF+ | - | Yes | Positive | Positive |
| 18 | Female | 24 | 161 | 58 | 22.4 | Non smoking | Upper lobe of left lung | No | BALF+ | 1+ | Yes | Positive | Positive |
| 19 | Female | 81 | 152 | 50 | 21.6 | Non smoking | Upper lobe of left lung | No | BALF+ | - | Yes | Positive | Negative |
| 20 | Female | 35 | 157 | 48 | 19.5 | Non smoking | Upper lobe of left lung | no | BALF+ | - | Yes | Positive | Negative |
| 21 | Female | 25 | 166 | 47 | 17.1 | Non smoking | Upper lobe of left lung | TB | lung biopsy+ | - | Yes | Positive | Positive |
| 22 | Female | 48 | 159 | 55 | 21.8 | Non smoking | Upper lobe of left lung | TB | BALF+ | - | Yes | Positive | Positive |
| 23 | Female | 32 | 155 | 42 | 17.5 | Non smoking | Lower lobe of left lung | no | BALF+ | 1+ | Yes | Positive | Positive |
| 24 | Female | 25 | 172 | 53 | 17.9 | Non smoking | Lower lobe of left lung | no | BALF+ | - | Yes | Positive | Negative |
| 25 | Female | 58 | 164 | 52 | 19.3 | Non smoking | Lower lobe of left lung | TB | BALF- | - | Yes | Negative | Positive |
| 26 | Female | 29 | 170 | 72 | 24.9 | Non smoking | Upper lobe of right lung | no | BALF- | - | No |  |  |
| 27 | Female | 34 | 162 | 42 | 16.0 | Non smoking | Upper lobe of right lung | no | BALF- | - | No |  |  |
| 28 | Female | 72 | 170 | 66 | 22.8 | Non smoking | Middle lobe of right lung | no | BALF- | - | No |  |  |
| 29 | Female | 61 | 165 | 71 | 26.1 | Non smoking | Upper lobe of right lung | no | BALF- | - | No |  |  |
| 30 | Female | 64 | 156 | 50 | 20.5 | Non smoking | Lower lobe of right lung | no | BALF- | - | No |  |  |
| 31 | Female | 30 | 159 | 55 | 21.8 | Non smoking | Upper lobe of right lung | no | BLAF- | - | No |  |  |
| 32 | Female | 19 | 166 | 42 | 15.2 | Non smoking | Lower lobe of right lung | no | lung biopsy- | - | No |  |  |
| 33 | Female | 63 | 162 | 57 | 21.7 | Non smoking | Lower lobe of right lung | no | BLAF- | - | No |  |  |
| 34 | Female | 34 | 160 | 60 | 23.4 | Non smoking | Lower lobe of left lung | no | BALF- | - | No |  |  |
| 35 | Female | 58 | 162 | 52 | 19.8 | Non smoking | Lower lobe of right lung | no | BALF- | - | No |  |  |
| 36 | Female | 43 | 160 | 55 | 21.5 | Non smoking | Lower lobe of right lung | no | lung biopsy- | - | No |  |  |
| 37 | Female | 78 | 150 | 36 | 16.0 | Non smoking | Middle lobe of right lung | no | BALF- | - | No |  |  |
| 38 | Female | 65 | 165 | 41 | 15.1 | Non smoking | Upper lobe of right lung | no | BALF- | - | No |  |  |
| 39 | Female | 57 | 160 | 75 | 29.3 | Non smoking | Lower lobe of left lung | no | BALF- | - | No |  |  |
| 40 | Female | 68 | 168 | 56 | 19.8 | Non smoking | Upper lobe of right lung | no | BLAF- | + | No |  |  |
| 41 | Female | 70 | 160 | 55 | 21.5 | Non smoking | Upper lobe of right lung | no | BALF- | - | No |  |  |
| 42 | Female | 57 | 158 | 38.5 | 15.4 | Non smoking | Upper lobe of right lung | no | BALF- | - | No |  |  |
| 43 | Female | 30 | 165 | 41 | 15.1 | Non smoking | Upper lobe of right lung | no | lung biopsy- | - | No |  |  |
| 44 | Female | 18 | 155 | 42 | 17.5 | Non smoking | Lower lobe of right lung | no | BALF- | - | No |  |  |
| 45 | Female | 44 | 165 | 60 | 22.0 | Non smoking | Lower lobe of left lung | no | BALF- | - | No |  |  |
| 46 | Female | 48 | 158 | 58 | 23.2 | Non smoking | Upper lobe of right lung | no | BALF- | - | No |  |  |
| 47 | Female | 56 | 165 | 70 | 25.7 | Non smoking | Lower lobe of right lung | no | BALF- | - | No |  |  |
| 48 | Female | 69 | 159 | 58 | 22.9 | Non smoking | Lower lobe of left lung | no | lung biopsy- |  | No |  |  |
| 49 | Female | 68 | 156 | 58 | 23.8 | Non smoking | Middle lobe of right lung | no | BALF- | - | No |  |  |
| 50 | Female | 70 | 156 | 55 | 22.6 | Non smoking | Middle lobe of right lung | no | BALF- | - | No |  |  |
| 51 | Female | 14 | 155 | 60 | 25.0 | Non smoking | Lower lobe of right lung | no | BALF- | - | No |  |  |

| **Clinical characterize of 56 positive T-SPOT patients included.** | | | | | | | | | | | | | |
| --- | --- | --- | --- | --- | --- | --- | --- | --- | --- | --- | --- | --- | --- |
| 1 | Male | 35 | 165 | 45 | 16.5 | Non smoking | Hilum pulmonale | Yes | BALF+ | 2+ | Yes | Positive | Positive |
| 2 | Male | 22 | 176 | 60 | 19.4 | Smoking | Upper lobe of right lung |  | BALF+ | 3+ | Yes | Positive | Positive |
| 3 | Male | 60 | 164 | 52 | 19.3 | Smoking | Upper lobe of right lung |  | lung biopsy+ | - | Yes | Positive | Negative |
| 4 | Male | 43 | 170 | 59 | 20.4 | Non smoking | Upper lobe of right lung | Yes | lung biopsy- | - | Yes | Positive | Positive |
| 5 | Male | 43 | 166 | 71 | 25.8 | Non smoking | Upper lobe of right lung | Yes | lung biopsy+ | - | Yes | Positive | Positive |
| 6 | Male | 76 | 160 | 40 | 15.6 | Non smoking | Upper lobe of right lung | Yes | lung biopsy+ | - | Yes | Positive | Positive |
| 7 | Male | 80 | 180 | 52.5 | 16.2 | Non smoking | Upper lobe of right lung |  | BALF- | + | Yes | Positive | Positive |
| 8 | Male | 38 | 171 | 64 | 21.9 | Smoking | Lower lobe of right lung | Yes | BALF+ | - | Yes | Positive | Positive |
| 9 | Male | 38 | 180 | 73 | 22.5 | Non smoking | Lower lobe of right lung |  | BALF+ | - | Yes | Positive | Negative |
| 10 | Male | 42 | 170 | 80 | 27.7 | Non smoking | Lower lobe of right lung |  | BALF+ | - | Yes | Positive | Positive |
| 11 | Male | 51 | 172 | 72 | 24.3 | Smoking | Lower lobe of right lung | Yes | lung biopsy+ | - | Yes | Positive | Positive |
| 12 | Male | 52 | 172 | 66 | 22.3 | Smoking | Middle lobe of right lung | Yes | lung biopsy+ | - | Yes | Positive | Positive |
| 13 | Male | 22 | 180 | 70 | 21.6 | Non smoking | Middle lobe of right lung | Yes | BALF+ | + | Yes | Positive | Positive |
| 14 | Male | 23 | 183 | 87 | 26.0 | Non smoking | Middle lobe of right lung | Yes | BALF+ | 2+ | Yes | Positive | Positive |
| 15 | Male | 56 | 172 | 60 | 20.3 | Smoking | Upper lobe of left lung |  | BALF+ | - | Yes | Positive | Negative |
| 16 | Male | 56 | 172 | 59 | 19.9 | Smoking | Upper lobe of left lung |  | BALF+ | - | Yes | Positive | Negative |
| 17 | Male | 33 | 180 | 69 | 21.3 | Non smoking | Upper lobe of left lung |  | BALF+ | - | Yes | Positive | Negative |
| 18 | Male | 50 | 177 | 75 | 23.9 | Smoking | Lower lobe of left lung | No | BALF+ | 3+ | Yes | Positive | Positive |
| 19 | Male | 22 | 175 | 56 | 18.3 | Non smoking | Lower lobe of left lung |  | BALF- | - | Yes | Negative | Negative |
| 20 | Male | 56 | 173 | 55 | 18.4 | Non smoking | Lower lobe of left lung |  | BALF+ | - | Yes | Positive | Positive |
| 21 | Male | 54 | 178 | 50 | 15.8 | Non smoking | Lower lobe of left lung | No | lung biopsy+ | - | Yes | Positive | Negative |
| 22 | Male | 24 | 173 | 68 | 22.7 | Non smoking | Upper lobe of left lung | Yes | lung biopsy- | - | No |  |  |
| 23 | Male | 26 | 180 | 130 | 40.1 | Non smoking | Lower lobe of left lung |  | BALF- | - | No |  |  |
| 24 | Male | 33 | 176 | 106 | 34.2 | Non smoking | Lower lobe of left lung | No | lung biopsy- |  | No | 随访抗结核治疗无效 | |
| 25 | Male | 33 | 185 | 92 | 26.9 | Non smoking | Lower lobe of right lung |  | BALF- | - | No |  |  |
| 26 | Male | 34 | 172 | 70 | 23.7 | Non smoking | Upper lobe of left lung | Yes | lung biopsy- |  | No |  |  |
| 27 | Male | 39 | 174 | 62 | 20.5 | Non smoking | Lower lobe of right lung |  | BALF- | - | No |  |  |
| 28 | Male | 40 | 170 | 70 | 24.2 | Non smoking | Upper lobe of left lung |  | BALF- | - | No |  |  |
| 29 | Male | 46 | 167 | 59.5 | 21.3 | Smoking | Upper lobe of right lung |  | BALF- | - | No |  |  |
| 30 | Male | 47 | 178 | 62 | 19.6 | Non smoking | Lower lobe of right lung |  | BALF- | - | No |  |  |
| 31 | Male | 50 | 170 | 65 | 22.5 | Non smoking | Middle lobe of right lung |  | lung biopsy- | - | No |  |  |
| 32 | Male | 52 | 168 | 65 | 23.0 | Non smoking | Middle lobe of right lung | No | BALF+ | - | No |  |  |
| 33 | Male | 54 | 168 | 73 | 25.9 | Non smoking | Lower lobe of right lung | No | BALF- | - | No |  |  |
| 34 | Male | 55 | 175 | 73 | 23.8 | Smoking | Upper lobe of left lung | No | lung biopsy+ | - | No |  |  |
| 35 | Male | 55 | 173 | 65 | 21.7 | Smoking | Upper lobe of left lung | No | BALF- |  | No |  |  |
| 36 | Male | 60 | 165 | 50 | 18.4 | Smoking | Middle lobe of right lung |  | BALF- | - | No |  |  |
| 37 | Male | 60 | 158 | 49 | 19.6 | Non smoking | Middle lobe of right lung |  | BALF- | - | No |  |  |
| 38 | Male | 60 | 178 | 81 | 25.6 | Non smoking | Lower lobe of right lung | No | BALF- | - | No |  |  |
| 39 | Male | 63 | 168 | 64 | 22.7 | Non smoking | Upper lobe of left lung |  | BALF- | - | No |  |  |
| 40 | Male | 64 | 165 | 70 | 25.7 | Smoking | Upper lobe of right lung | No | lung biopsy- | - | No |  |  |
| 41 | Male | 65 | 174 | 83 | 27.4 | Smoking | Upper lobe of left lung | No | lung biopsy- | - | No |  |  |
| 42 | Male | 65 | 172 | 61 | 20.6 | Smoking | Lower lobe of right lung |  | BALF- | - | No |  |  |
| 43 | Male | 68 | 168 | 60 | 21.3 | Smoking | Lower lobe of right lung | No | BALF- | - | No |  |  |
| 44 | Male | 68 | 173 | 77 | 25.7 | Smoking | Lower lobe of left lung |  | BALF- | - | No |  |  |
| 45 | Male | 81 | 170 | 67 | 23.2 | Non smoking | Upper lobe of left lung | No | lung biopsy- | - | No |  |  |
| 46 | Male | 85 | 165 | 55 | 20.2 | Smoking | Lower lobe of left lung |  | BALF- | - | No |  |  |
| 47 | Male | 31 | 171 | 85 | 29.1 | Non smoking | Lower lobe of right lung | No | BALF- | - | No |  |  |
| 48 | Male | 68 | 160 | 51.5 | 20.1 | Smoking | Upper lobe of right lung | No | BALF- | - | No |  |  |
| 49 | Male | 71 | 176 | 73 | 23.6 | Smoking | Lower lobe of right lung | No | BALF- | - | No |  |  |
| 50 | Male | 63 | 170 | 65.5 | 22.7 | Smoking | Upper lobe of right lung | No | lung biopsy- |  | No |  |  |
| 51 | Male | 59 | 172 | 80 | 27.0 | Non smoking | Upper lobe of right lung |  | BALF- | - | No |  |  |
| 52 | Male | 57 | 168 | 84 | 29.8 | Smoking | Upper lobe of right lung | No | BALF- | - | No |  |  |
| 53 | Male | 55 | 165 | 58 | 21.3 | Smoking | Lower lobe of right lung | No | lung biopsy- | - | No |  |  |
| 54 | Male | 79 | 160 | 52 | 20.3 | Smoking | Upper lobe of right lung | No | lung biopsy- |  | No |  |  |
| 55 | Male | 73 | 162 | 51 | 19.4 | Smoking | Lower lobe of left lung | No | BALF- | - | No |  |  |
| 56 | Male | 44 | 158 | 60 | 24.0 | Non smoking | Lower lobe of left lung |  | BALF- | - | No |  |  |
